# Supplementary material for: Polyketide synthase gene clusters in the ericoid mycorrhizal fungus Oidiodendron maius: genome-wide prediction, expression profiling, and functional characterization of knockout mutants
Source: Front Plant Sci. 2026 Sep 16;17:1924430. doi: 10.3389/fpls.2026.1924430 (PMC13623903; doi:10.3389/fpls.2026.1924430)
Supplement: Supplementary file 1 [file SupplementaryFile1.zip › Supplementary Materials/Supplementary Folder 1.zip!/Oidma1_AssemblyScaffolds_Repeatmasked/knownclusterblast/cluster49/jgi.p_Oidma1_35134_mibig_hits.html]

| MIBiG Protein | Description | MIBiG Cluster | MiBiG Product | % ID | % Coverage | BLAST Score | E-value |
| --- | --- | --- | --- | --- | --- | --- | --- |
| AHB62772.1 | AmbI2 | BGC0001126 | Ambiguine\_biosynthetic\_gene\_cluster | 28 | 58.3673469388 | 123 | 7.3e-28 |
| AHI58840.1 | WelI2 | BGC0000668 | Welwitindolinone\_biosynthetic\_gene\_cluster | 27 | 61.6326530612 | 119 | 1.4e-26 |
